# Probing local and electronic structure in Warm Dense Matter: single pulse synchrotron x-ray absorption spectroscopy on shocked Fe

**DOI:** 10.1038/srep26402

**Published:** 2016-06-01

**Authors:** Raffaella Torchio, Florent Occelli, Olivier Mathon, Arnaud Sollier, Emilien Lescoute, Laurent Videau, Tommaso Vinci, Alessandra Benuzzi-Mounaix, Jon Headspith, William Helsby, Simon Bland, Daniel Eakins, David Chapman, Sakura Pascarelli, Paul Loubeyre

**Affiliations:** 1 CEA, DAM, DIF, F-91297, Arpajon, France; 2 ESRF-The European Synchrotron 71, Avenue des Martyrs Grenoble, France; 3 LULI - CNRS, Ecole Polytechnique, CEA: Université Paris-Saclay, France; 4 UPMC Univ Paris 06:Sorbonne Universités - F-91128 Palaiseau cedex, France; 5 Science and Technology Facilities Council, Daresbury Laboratory Sci-Tech Daresbury, Warrington WA4 4AD, UK; 6 Institute of Shock Physics, Imperial College London, London SW7 2AZ, UK.

## Abstract

Understanding Warm Dense Matter (WDM), the state of planetary interiors, is a new frontier in scientific research. There exists very little experimental data probing WDM states at the atomic level to test current models and those performed up to now are limited in quality. Here, we report a proof-of-principle experiment that makes microscopic investigations of materials under dynamic compression easily accessible to users and with data quality close to that achievable at ambient. Using a single 100 ps synchrotron x-ray pulse, we have measured, by K-edge absorption spectroscopy, ns-lived equilibrium states of WDM Fe. Structural and electronic changes in Fe are clearly observed for the first time at such extreme conditions. The amplitude of the EXAFS oscillations persists up to 500 GPa and 17000 K, suggesting an enduring local order. Moreover, a discrepancy exists with respect to theoretical calculations in the value of the energy shift of the absorption onset and so this comparison should help to refine the approximations used in models.

## Introduction

Warm Dense Matter refers to a material state that occurs, under extreme thermodynamic conditions, at the intersection of condensed matter physics and plasma physics^1^. The limits of WDM are not sharply defined: it is the region where electron degeneracy, ion coupling and atomic physics all play a role in the material response, i.e. typically density and temperature are in units of solid density and 10^4 ^K (~eV) respectively. The most advanced calculations of WDM are made using first principle density functional theory^2^,^3^. It is a ground state electronic theory that has been very successfully extended to predict the properties of compressed matter at ambient temperature but the approximations for its extension in the eV temperature range, where the contributions of excited electronic states are important, still need to be validated.

Unfortunately, very little experimental data exist to test the predictions of these calculations at the atomic level. The equation of state of WDM is currently obtained at dynamic compression facilities (such as multi-stage gas guns, multi-mega ampere pulsed power generators and high energy lasers) by measuring the shock velocities. The link between the measured temperature –pressure – density relation and the microscopic state and atomic structure is the next step to progress in the validation of WDM models. Over the last decade, much effort has been devoted to the development of *in-situ* x-ray probes for atomic scale characterization of compressed matter in front of these various dynamic facilities. Most common techniques use x-ray back-lighters generated by lasers. Recently, significant results were achieved using x-ray absorption, on ramp compressed iron to around 500 GPa^4^ in the EXAFS (Extended X-ray Absorption Fine Structure) range, on aluminum shocked to 8 eV^5^ and SiO_2_ to 5 eV^6^ in the XANES (X-ray Absorption Near Edge Spectroscopy) range. However, these x-ray characterizations have not reached the energy resolution and level of detail achievable on synchrotron beamlines, even for samples under high static pressure-temperature conditions^7^.

Here, we propose an alternative line of progress by combining the best possible x-ray characterization methods available at synchrotrons to dynamic compression^8^,^9^. This is based on a similar strategy as the one used by the static high pressure community, namely minimizing the volume of the compressed sample to achieve extreme pressures and take full advantage of the high brightness (10^14^ photons/s/10 μm^2^), extreme stability (fluctuations of normalized intensity ≤0.002 at ~1 KHz frequency), high energy resolution (δE/E ~ 10^−4^), large spectral range (ΔE/E > 10%) and small spot size (typically less than 10 μm^2^) of the synchrotron x-ray beam. The shocked states were generated by a portable 35 J laser. The laser pulse was focused to a diameter of ~90 μm (high power density configuration) or ~350 μm (low power density configuration), resulting in similar compressions as those obtained by kJ lasers on mm size samples. Measurements were performed at the energy dispersive x-ray absorption beamline ID24^10^ of the European Synchrotron Radiation Facility, which has been recently upgraded. X-ray absorption fine structure, which includes XANES and EXAFS, is an element selective probe of the local electronic and atomic structure. Hence, it is well adapted for the characterization of WDM at the atomic level. Iron is a key constituent of terrestrial and exoplanets. Given its ubiquitous importance, Fe is often one of the first materials to be investigated with novel experimental techniques. With this in mind, the present data are thus compared to XAS measurements recently performed under dynamic compression at the OMEGA laser^4^ and LCLS free electron laser facilities^11^.

## Results

**Figure 1:**
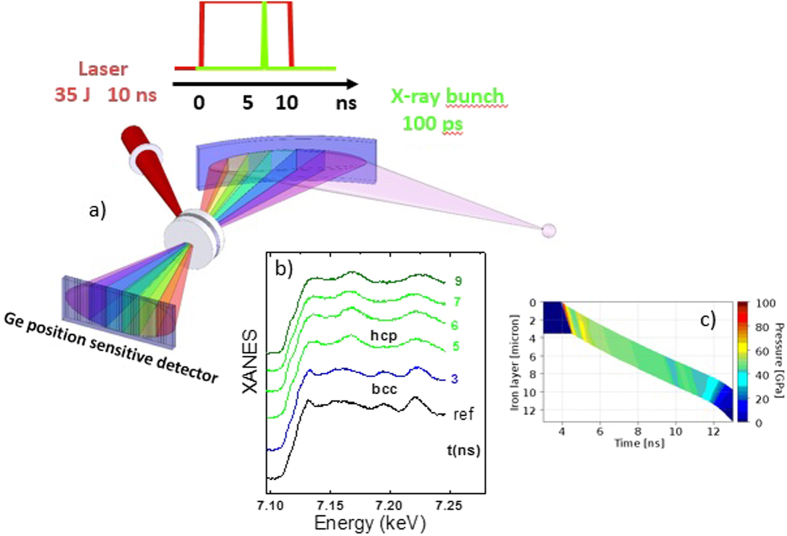
Schematic view of the experimental set-up. Panel (**a**) a curved Si-crystal focuses the polychromatic x-ray beam onto the sample. The beam transmitted by the sample diverges towards a position sensitive detector where energy is correlated to position; in this way, the full EXAFS spectrum is acquired at once by a position sensitive detector with Ge pixels (1024). A long laser pulse (10 ns, up to 35 J, at 1057 nm) focused at the sample position, drives the compression wave in Fe. The Fe target consists of a 3.5 μm iron layer sandwiched between two diamond windows. Panel (**b**) a series of typical single bunch XANES spectra obtained by changing the x-ray probe delay time with respect to the laser onset time, while keeping the same driving energy for each shot, shows that the compressed state is in the hcp phase and its thermodynamic conditions are stable over 2 ns at least. Panel (**c**) 1-D hydro-simulation of the shock in the Fe layer with a similar color scale for the pressure as for the XANES spectra.

The sketch of the experimental configuration is presented in Fig. 1 panel a. In the dispersive geometry, a fan of x-rays is dispersed and focused on the sample by a curved crystal, so that the energy of each ray varies as a function of convergence angle. The transmitted photons are then detected by a position sensitive detector allowing the simultaneous collection of the whole spectrum, up to a 300 eV range above the Fe K-edge (7.112 keV). Data were acquired using a single x-ray pulse of 100 ps duration, during a 4-bunch mode configuration of the synchrotron storage ring that assures the highest number of photons per pulse and a maximum separation between pulses (700 ns). The x-ray spot could be focused down to 5 μm × 7 μm FWHM (H × V) to ensure that the probed part of the compressed sample was not perturbed by edge effects. The position sensitive detector equipped with 1024 Ge pixels was operated with an integration time of 680 ns and phase locked to the ESRF radiofrequency system, ensuring integration of the signal generated by a unique bunch of electrons in the storage ring. The absorption spectrum so acquired for the undriven sample (in Fig. 1, panel b, the *ref* spectrum) shows fine structure features comparable to those of a spectrum acquired on an energy scanning XAS beamline. The <35 J energy of a 1057 nm laser delivered in a 10 ns square pulse was focused under vacuum on the target with an angle of 30°, launching an ablation driven compression in the target perpendicular to its surface. The x-ray beam probed the shocked Fe parallel to the compression axis with variable drive-probe delay.

The minimization of the compressed volume of Fe required a careful target design that was optimized using two 1D hydro-codes, ESTHER^12^ and MULTI^13^. The target was made of a thin (3.5 μm) deposit of pure Fe sandwiched between two diamond windows, which act to confine the shocked state over a time interval much longer than the 100 ps x-ray pulse. A CH layer was deposited on the ablation surface to limit pre-heating effects. Two different kinds of targets were prepared for the two laser configurations used to cover a large thermodynamic range. In the first case, diamond windows of 40 (front) and 50 (rear) μm were used and the laser was focused with a phase plate of ~350 μm producing a maximum usable ablation laser intensity of 4 × 10^12 ^W/cm^2^ and so a maximum pressure of ~80 GPa in Fe. The x-ray focal spot was adjusted to 5 × 100 μm (H × V) FWHM in this configuration. The shock launched in the front window generates reverberating compression waves in the iron foil due to the presence of the rear diamond window. The pressure reached in Fe is approximately the pressure of the diamond front window and because of the impedance matching between diamond and iron, the final state of iron is near its principal Hugoniot. A 2-D simulation with the DUED code^14^ was performed to evaluate rarefaction edge effects and the homogeneous compressed region in Fe was calculated to be 200 μm in diameter. The confinement time of the stable thermodynamic conditions in Fe was estimated by hydrodynamic simulations and measured directly from the modifications in the XANES spectra for various delays between the laser arrival on the target and the x-ray pulse. As seen in Fig. 1 panel b, a 4 ns confinement time is observed, in agreement with the hydro-simulations (panel c); within this interval, thermodynamic conditions are stable for at least 2 ns. The pressure was estimated by hydro-simulations using MULTI^13^ and ESTHER^12^ codes and also double-checked using an empirical law giving the ablation pressure versus the laser intensity in the ablator^15^ (see the Supplementary material); the error in the pressure is estimated as the difference between the two hydrodynamic codes outputs.

The reproducibility of the measured spectra was good. We are thus confident that homogeneous well defined compressed states of Fe were measured.

**Figure 2:**
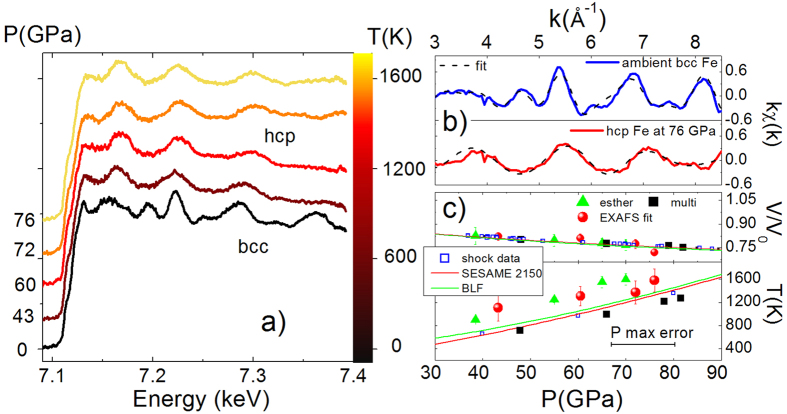
EXAFS spectra, volume and temperature determination. Panel (**a**) Series of EXAFS spectra obtained by increasing the irradiation intensity at 7 ns of delay, driving the compression in the 350 μm phase plate configuration. The spectra under shock are compared to the reference spectra at ambient. The structural change between the bcc and hcp phase is clearly seen. Panel (**b**) Fit of the EXAFS for the reference spectrum and the most compressed hcp one. The compression factor and the temperature extracted for each intensity are indicated in panel (**c**) with comparison to the simulations outputs, literature Hugoniot curves^27^,^28^ and shock data^25^,^26^. The pressure value for the EXAFS points is given by the average between the two hydrodynamic codes outputs.

The evolution of the K-edge EXAFS spectra at different pressures is shown in Fig. 2 (panel a); no deterioration of the spectra quality between the undriven and the shocked states is observed. The ambient spectrum was modeled with the bcc structure and those at higher pressure, from 40 GPa, with the hcp structure, as suggested by the changes in the shape of the spectral features. For example, the disappearance of the peak around 7.2 keV is the signature of the bcc-hcp transition^16^. A quantitative analysis of the EXAFS spectra enabled the determination of the volume and temperature of the compressed solid states^4^,^17^ (Fig. 2 panel c) . The fits of normalized EXAFS signals, χ(k), of the unshocked and highest pressure shocked samples are shown in panel b of Fig. 2. The fit allows the lattice parameters *a*_bcc_ and *a*_hcp_ to be determined, and thus the volume (*V*_bcc _= *a*^3^/2 and *V*_hcp _= 0.433 *a*^2^ *c*). In the hcp phase, *c/a* was fixed to the value 1.61 since the k-range of the EXAFS was not sufficient for its precise adjustment; however, simulations and static measurements^18^,^19^,^20^,^21^,^22^ have shown that the *c*/*a* value remains almost ideal (1.59–1.63) over the thermodynamic region covered here. The temperatures were also estimated from the EXAFS spectra using a correlated Debye model^23^, where the Debye temperature as a function of compression was taken from ref. 24 (see the Methods session for further details on the fitting procedures). In panel c we compare V and T values obtained from the EXAFS analysis to the hydrodynamic codes outputs, literature EoS^27^,^28^ and shock data^25^,^26^. The compression data obtained by the EXAFS analysis are found quite close to previous shock data^25^,^26^, EoS^27^,^28^ and simulations values. The EXAFS temperature values are in agreement with the outputs from the ESTHER code, systematically higher than the Hugoniots. The difference found in the PT and PV outputs from the two codes origins from the different parameters and models used in the calculations and is a good estimation of the error made by this kind of calculations (see the Supplementary material for further details).

**Figure 3:**
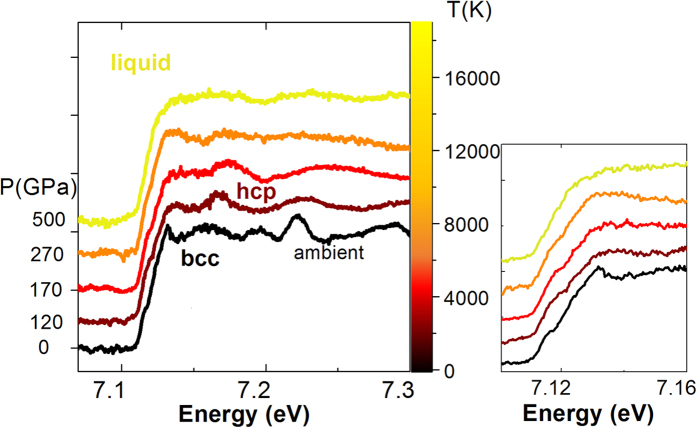
EXAFS spectra and structural changes. (Left) A series of EXAFS spectra obtained in the highly focused configuration of the laser. (Right). Zoom over the edge region.

In order to achieve pressures in the 500 GPa range, the 350 μm phase plate was removed and the laser directly focused to a 90 μm spot with a Gaussian spatial profile. To maintain a homogeneous thermodynamic state and limit 2D effects, Fe targets with 25 μm thick diamond windows were used in that case. 2D-simulations give a 40 μm diameter homogeneous region, larger than the x-ray probe beam. An additional vertical focusing mirror was used to reduce the x-ray focal spot down to 5 × 7 μm (H × V) FWHM. Again, a homogeneous thermodynamic state could be probed, however, the reproducibility from shot to shot was poorer due to the request in alignment of a 10 μm precision coupled to the lower spatial homogeneity of the laser profile. As seen in Fig. 3, the EXAFS data quality obtained in this second configuration was not as good as for the spectra acquired with the phase plate and with a larger x-ray spot (the signal to noise ratio decreased by a factor ~2). The high frequency noise is related to lower photon statistics, because the additional mirror could not be put under vacuum. However, as seen in Fig. 3, clear trends can be followed in the spectra as a function of the laser intensity (hence pressure), both in the XANES and in the first EXAFS oscillations region. The compression and heating trend are revealed from the shift to higher energy and the broadening of the EXAFS oscillations respectively (black, purple and red spectra in Fig. 3). As pressure increases this trend is broken (orange spectrum) and the bump in the edge (at E = 7.12) starts to flatten, indicating the onset of melting, in agreement with the recent melting curve from Anzellini *et al.*^18^. The most extreme shocked state has been achieved with I = 5 × 10^13 ^W/cm^2^, with a corresponding pressure estimated around 500 GPa. Remarkably, weak EXAFS oscillations remain as liquid Fe is further compressed and heated to such an extreme state, indicating a persisting local order in WDM.

**Figure 4:**
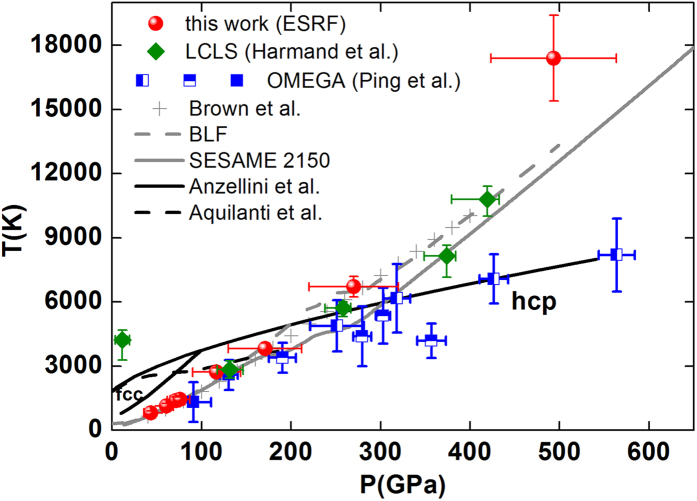
Iron phase diagram. Phase diagram of Fe showing our data (red full circles) in comparison to recent data from the literature. Full green triangles are from Harmand *et al.*^11^; blue squares are from Ping *et al.*^4^: full is single shock, half horizontal is multiple shock with P_0 _= 100 GPa and half vertical is multiple shock with P_0 _= 150 GPa; black lines are melting lines from ref. 18 (full) and ref. 7 (dash). Grey curves indicate different estimations of Fe Hugoniot, from Brown^25^, SESAME^27^ and BLF^28^.

Figure 4 shows the states in the phase diagram of Fe that have been probed by XAS measurements under dynamic compression, namely our data (red full circles) and measurements on two other platforms^4^,^11^. Although the thermodynamic domains of investigation are comparable in these three experiments, the use of the synchrotron facility enables to improve the XAS data. The experiment^11^ at the x-ray free electron laser LCLS could not bring much insight on the local order in the liquid phase. The energy range (25 eV) was too narrow to cover even the first EXAFS oscillations and the data quality suffered from the intrinsic intensity fluctuations of the incident beam, leading to the necessity to average over few tens of shots to acquire satisfactory spectra. The second experiment^4^ at the OMEGA facility produced very good quality EXAFS data of Fe compressed to 560 GPa but the XANES region could not be exploited due to a lack in energy resolution^29^.

The present P T data points are close to the Fe principal Hugoniot curve up to around 270 GPa and 6700 K (corresponding to a laser power of 3 × 10^13 ^W/cm^2^) where the hydrodynamic simulations start to witness some preheating which makes the two last points higher in temperature (see the Supplementary material for further details on the simulations). Our data agree with the melting curve of Anzellini *et al.*^18^: our point at 170 GPa and 3800 K (red spectrum in Fig. 3), which is close to the new melting curve of Aquilanti *et al.*^7^ still shows a pronounced bump at the absorption edge, a signature of the solid phase. However, a precise determination of the melting curve is beyond the scope of this paper.

**Figure 5:**
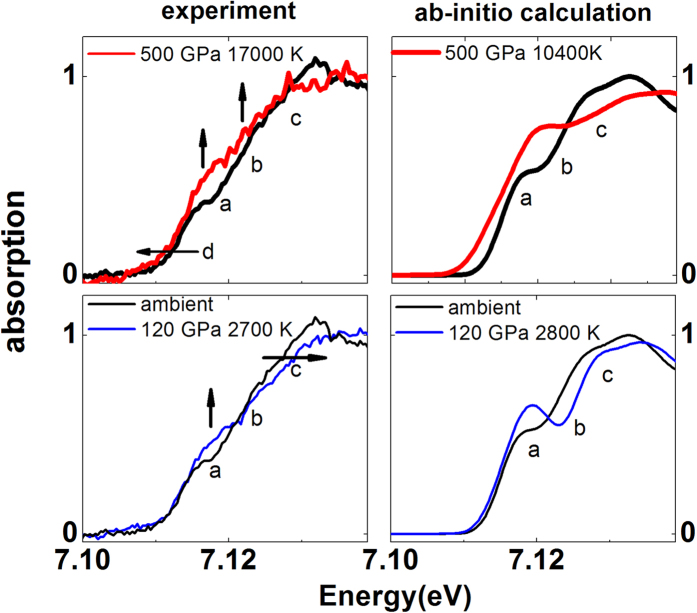
Evolution of the absorption at the K-edge onset. Left: zoom over the edge region of a selection of spectra acquired in the highly focused configuration of the laser. a, b, c and d indicate regions where major changes are observed. Right: ab-initio molecular dynamic simulations (based upon private communication with Vanina Recoules from refs 3 and 11).

The evolution of the absorption at the K-edge of iron is shown in Fig. 5. The quality of the data is sufficient to detect the evolution of features (noted *a*, *b*, *c*, *d*) as predicted by ab-initio molecular dynamics calculations^3^,^11^ and as observed in the laser heated DAC^7^. In the compressed hcp solid (blue spectrum in Fig. 5 left bottom panel) feature *a* increases in intensity and feature *c* moves to higher energy due to the compression. At higher drive intensity up to I = 5 × 10^13 ^W/cm^2^, corresponding to around 500 GPa and 17000 K, feature *c* moves back to lower energies and feature *a* increases more in intensity as the edge is modified into a rounded shape: such drastic change is the sign of a molten phase. In fact, similar behavior has been recently observed in lower pressure laser heating experiments (<100 GPa) probing the melting of Fe^7^,^30^. By comparing the available ab-initio molecular dynamics simulations of iron in similar conditions (Fig. 5 right panels) to our data, we observe that although the shape of the absorption onset is more structured than the experimental one, theory is capable of grasping the main changes in the data (indicated with arrows). However, whereas good agreement between the theoretical prediction and the data is observed in the energy shift of the absorption onset (7.112 keV, feature *d*) for the compressed solid (Fig. 5, bottom panels), an important discrepancy is found for the data recorded at the highest P, T values (Fig. 5, top panels): the theoretical shift is ~ −2 eV, whereas the experimental shift is ~ −0.5 eV.

## Discussion

In the low power density configuration, the volume and the temperature could be extracted from the fit of the EXAFS oscillations. In the high power density configuration, the temperature induced broadening of the EXAFS oscillations does not enable volume extraction and local order structural refinement. However, a clear signature of the hcp structure could be followed up to the melting of Fe. This observation confirms the great stability of the hcp structure in Fe under very high pressure, as shown previously utilizing static laser heated DAC^18^ or the dynamic multi shock compression^4^.

At the most extreme states achieved (500 GPa and 17000 K), the electrons degeneracy factor is around 2 and the plasma coupling parameter around 6, and so thermodynamic conditions for Fe enter into the WDM regime^1^. With further optimization of the technique, future measurements are expected to yield data up to 500 GPa and above of similar quality as in Fig. 2, thus allowing a finer comparison to theoretical models and a possibility to disclose the local order in WDM Fe.

The coupling between local order and the electronic state is also an important issue for models of WDM. Changes in the features of the absorption edge indeed reflect this coupling. The general trend of the modification of the K-edge spectra along the Hugoniot is well reproduced by DFT calculations with the exception of the value of the shift of the edge at 500 GPa (0.5 eV measured and 2 eV calculated). Since the K-edge shift is related to the energy difference, E_F_-E_1s_ , between the Fermi energy and the 1s core energy level, it reflects the modification of the core energy level and of the electron density. In Fe, this K-edge shift along the Hugoniot is predicted to be much smaller than in Al^5^, in line with our observation. In the Warm Dense regime, this shift is particularly sensitive to changes in electronic shielding due to ionization, and to continuum lowering depression. The XANES data therefore provide important constraints on the electronic treatment of WDM within the DFT framework.

In summary, we present data from a proof-of-principle experiment demonstrating that single pulse XAS synchrotron measurements will enable determination of structural and electronic properties of WDM with accuracy close to that on materials at ambient. Bringing the standard of synchrotron material studies towards the measurement of the atomic and microscopic properties of WDM will help validate models at the microscopic level and reveal new phenomena. We provide the first EXAFS data of Fe compressed to 500 GPa and heated to 17000 K, covering a region of the phase diagram not previously explored conclusively with x-ray diagnostics. Our data indicate a persistent local order in the dense Fe fluid. The experimentally measured value of the shift of the absorption edge at 500 GPa with respect to ambient is found to be smaller than the theoretical prediction, stimulating further work to improve the electronic treatment of WDM in the DFT framework. The present experimental approach could easily be extended to higher pressures and temperatures by using a more powerful (100–200 J) laser that would remain compatible with the beamline configuration.

## Methods

### Target preparation

The targets were made of a thin (3.5 μm) deposit of pure Fe sandwiched between two diamond windows, whose role was to confine the shock for a time interval much larger than the synchrotron pulse duration (100 ps). The Fe deposit was realized by the DEPHIS company and the Fe initial density was measured to be that of the solid (7.87 ± 0.08 g/cc). This was confirmed by the obtained x-ray absorption jump, which is dependent on the density. The adherent Fe deposit was realized on the rear window, a good contact to the ablation window was assured by an interstitial thin layer of CH (<1 μm) and by the windows holder specifically designed to “close” the sandwich by exerting a slight mechanical pressure. Diamond windows of around 40 μm (front) and 50 μm (rear) were used when the laser was focused to 350 μm with the phase plate, whereas thinner windows (25 μm) were used when the laser was focused down to 90 μm to limit the 2D effects. The lateral dimension of the windows, and thus of the Fe deposit was 2 mm, and the holder opening was 1.2 mm. A 4 μm layer of CH was deposited on the ablation face to limit the pre-heating effect.

### Laser, synchronization and alignment

The experiment was performed with the QUANTEL portable high power laser system provided by CEA. The maximum energy of the laser was 35 J, delivered in a square pulse of 10 ns. The diameter of the laser focal spot was around 350 μm and 90 μm with and without a phase plate respectively, as measured with a 30 degree incidence angle using a blade and sending the image to a Spiricon camera. The laser maximum repetition rate was 45 sec. All the laser clocks were locked to the ESRF radiofrequency system with the proper frequency divisions. The delay between the laser and the x-ray pulse was pre-aligned using a GaAs APD. A fine temporal adjustment was performed directly looking at the onset of the compressed hcp state in the sample evident through the changes in the XANES. The spatial alignment between the laser and the x-ray beam was performed using the following procedure. First the x-ray beam position was determined by scanning a reference target containing a metallic foil. We then used a high magnification camera looking at the front face of the target to visualize the laser at low power and bring it to the x-ray beam position. Since the laser spot size was always much bigger than the x-ray spot, the error in the alignment is given by the accuracy in the determination of the x-ray beam position which we estimate to be around 10 μm.

### Hydrodynamic simulation of the target

1D Hydrodynamic simulations using the Lagrangian ESTHER^12^ code were performed using a mesh of around 900 units to describe the diamond-Fe-diamond sandwich. The EOS for plastic and diamond were taken from SESAME tables 7592 and 7834 respectively while a BLF^28^ (Bushman, Lomonosov and Fortov) multiphase equation was used for iron. Opacities are given by the NOHEL code by A. Decoster and at low temperature by measurement from Henke^31^. The strength of diamond is taken into account by using a perfectly elastoplastic model, which includes a simple constitutive model where the elastic yield stress and the shear modulus are constant in the solid domain and vanish to zero when the diamond temperature reaches the melting temperature. The values of the elastic yield strength and shear modulus used to simulate the strength of diamond are 7.5 10^9^ (erg/cm^3^) and 4.77 10^12^ (erg/cm^3^) respectively.

The 1D Hydrodynamic simulations using the MULTI^13^ code were performed using a mesh of around 550 units for the thin targets and 800 for the thick targets. The SESAME 7830 was used for the diamond, and the SESAME 2150 for iron. Opacities are taken from a SNOP model^32^. In both cases thermal conductivity and radiative transfer were included in the calculation.

### EXAFS analysis

The EXAFS quantitative analysis was performed using the IFFEFIT package^33^ for the spectra up to laser intensities of 4 × 10^12 ^W/cm^2^. A five shells model was used for the fit to the ambient bcc spectrum; assuming an undistorted bcc structure, only one distance parameter was fitted. Temperature and Debye temperature were set to ambient values. The volume was then calculated as *V*_bcc _= *a*^3^/2. Other fitting parameters were the energy offset Δ*E* and the amplitude reduction factor *S*_*0*_^*2*^. The obtained value of *S*_*0*_^*2*^ = 0.7 ± 0.1 was then fixed in the fits to the compressed hcp structures and its error used to evaluate the temperature error; in fact, temperature and S_0_^2^ are strongly correlated, therefore the main source of error in the temperature determination comes from the uncertainty over the S_0_^2^. A two shells model was used for the hcp structure, assuming a homogeneous compression between the two, so that only one distance parameter was fitted. The fit allows the lattice parameter *a*_hcp_ and thus volume *V*_hcp _= 0.433 *a*^2^ *c* to be obtained. The *c/a* parameter was fixed to the value 1.61 since the k-range of the EXAFS was not extended enough for its precise adjustment; however simulations and static measurements^18^,^19^,^20^,^21^,^22^ have shown that the *c*/*a* value remains almost ideal (1.59–1.63) over the thermodynamic region of interest. Other fitting parameters were the energy shift Δ*E* and the temperature *T*. The k-range of the collected data was not sufficient for the analysis to include anharmonic corrections. However, ab-initio molecular dynamics simulations suggest that these are small in hcp Fe up to inner core conditions^22^. A correlated Debye model was used for the temperature fit^23^ which is appropriate in our temperature range^4^. The Debye temperature at different compressions in the hcp phase is taken from published measurements^24^.

## Additional Information

**How to cite this article**: Torchio, R. *et al.* Probing local and electronic structure in Warm Dense Matter: single pulse synchrotron x-ray absorption spectroscopy on shocked Fe. *Sci. Rep.* **6**, 26402; doi: 10.1038/srep26402 (2016).
